# Automated Detection of Streptococcus pyogenes Pharyngitis by Use of Colorex Strep A CHROMagar and WASPLab Artificial Intelligence Chromogenic Detection Module Software

**DOI:** 10.1128/JCM.00811-19

**Published:** 2019-10-23

**Authors:** Tam T. Van, Kenneth Mata, Jennifer Dien Bard

**Affiliations:** a Department of Pathology, Harbor-UCLA Medical Center, Torrance, California, USA; b Department of Pathology and Laboratory Medicine, David Geffen School of Medicine, Los Angeles, California, USA; c Department of Pathology and Laboratory Medicine, Children’s Hospital Los Angeles, Los Angeles, California, USA; d Keck School of Medicine, University of Southern California, Los Angeles, California, USA; Johns Hopkins University School of Medicine

**Keywords:** chromogenic detection module, group A *Streptococcus*, WASPLab

## Abstract

Colorex Strep A agar (CHROMagar, Paris, France) was evaluated with PhenoMATRIX chromogenic detection module (CDM) software (Copan Diagnostics Inc., Murrieta, CA) to detect group A *Streptococcus* (GAS) from throat specimens. The software results were compared to those of manual plate image reading. In addition, GAS PCR testing was performed on all specimens. True-positive specimens were defined as culture-positive (by either PhenoMATRIX CDM or manual reading) specimens confirmed as GAS by matrix-assisted laser desorption ionization–time of flight mass spectrometry plus any culture-negative specimens that were positive by both initial and repeat PCR testing. Of 480 specimens, 96 were considered true-positive specimens. Software reading of the chromogenic agar for suspected colonies detected 110 orange colonies, whereas technologist reading interpreted only 93/110 specimens (84.5%) as positive. None of the 361 cultures interpreted as negative by the PhenoMATRIX CDM software was positive by manual reading. In comparison with true-positive results, the sensitivity and specificity were 96.9% and 100% for PCR testing, 87.5% and 97.7% for technologist reading of chromogenic agar, 90.6% and 94.0% for software reading of chromogenic agar, 83.3% and 97.7% for technologist reading for β-hemolysis on blood agar, and 39.5% and 83.1% for technologist reading for β-hemolysis on blood agar accompanied by any zone of inhibition around a bacitracin-impregnated disk, respectively. The software had the most accurate results of the non-molecular testing methods, detecting all suspected colonies on the chromogenic agar and identifying 3 additional true-positive specimens that were missed by manual reading. The PhenoMATRIX CDM software and the Colorex Strep A agar can improve detection of GAS from throat specimens, and they compared favorably to molecular testing.

## Abstract (precis)

Colorex Strep A agar (CHROMagar, Paris, France) was evaluated with PhenoMATRIX chromogenic detection module (CDM) software (Copan Diagnostics Inc., Murrieta, CA) to detect group A *Streptococcus* (GAS) from throat specimens. The software results were compared to those of manual plate image reading. In addition, GAS PCR testing was performed on all specimens.

## INTRODUCTION

Pharyngitis is diagnosed in approximately 11 million patients in the United States each year (1). Although the vast majority of these cases are caused by viruses, Streptococcus pyogenes (group A *Streptococcus* [GAS]) is the most common bacterial cause of acute pharyngitis, accounting for 15 to 30% of cases in children and 5 to 10% of cases in adults (2–4). During the winter and spring in temperate climates, up to 20% of asymptomatic school-aged children may be GAS carriers and vessels for infecting others (5). Signs and symptoms of GAS pharyngitis overlap extensively with those of other infectious causes, and it can be difficult to differentiate between bacterial and viral causes (6).

To assist in the timely differentiation between streptococcal pharyngitis and other causes, a number of laboratory tests to detect GAS are available, ranging from direct antigen detection to newer direct molecular assays. However, the American Academy of Pediatrics continues to recommend that negative rapid antigen test results and some molecular assay results be confirmed for children using the gold standard of throat culture, unless the assays have been shown to have sensitivity similar to that of throat culture (7). Throat cultures have high sensitivity, compared to some rapid antigen tests, and a lower cost than molecular methods. The use of culture for pharyngitis in large-volume laboratories can be cumbersome and would benefit from a more streamlined approach using automated plating instrumentation, smart incubation, and image analysis to differentiate and to segregate suspected positive cultures. Previous studies using automatic digital analysis of chromogenic agar by the WASPLab PhenoMATRIX chromogenic detection module (CDM) have accurately identified vancomycin-resistant enterococci and methicillin-resistant Staphylococcus aureus (MRSA) (8, 9).

This study evaluated the capability of the Copan WASPLab PhenoMATRIX artificial intelligence CDM software to automatically detect and to interpret GAS colonies on the new chromogenic agar, Colorex Strep A agar. The chromogenic agar and software results were compared to manual plate reading by medical technologists. The Lyra Direct Strep PCR assay (Quidel Corp., San Diego, CA) was also performed for all specimens.

## MATERIALS AND METHODS

### Clinical specimen enrollment.

A total of 480 pharyngeal samples, collected during the period of September 2017 through January 2019 from pediatric patients presenting to the emergency department at Children’s Hospital Los Angeles (CHLA) with presumed bacterial pharyngitis, were enrolled in this study. CHLA is a freestanding, academic, tertiary care, pediatric institution with a large emergency department that serves a large diverse pediatric community. This study was reviewed and approved by the CHLA institutional review board (IRB) (IRB protocol CHLA-17-00416).

Samples were collected using the ESwab transport system (Copan Diagnostics Inc., Murrieta, CA) and initially tested for the presence of GAS by the Lyra Direct Strep PCR assay (Quidel), in the clinical microbiology laboratory at CHLA. The Lyra Direct Strep assay detects and differentiates GAS from groups C and G but does not differentiate between groups C and G. The Lyra assay was performed as the standard of care, following the manufacturer’s recommendations, and its performance was previously determined at CHLA (10). Remnant ESwab samples were frozen at –70°C after standard-of-care testing was completed; all additional testing was performed within 45 days after the initial PCR testing except for 2 samples, which were tested >12 months later.

### Culture examination.

Frozen samples were transferred to another facility, where they were inoculated onto blood agar plates (BAPs) and Colorex Strep A agar (CHROMagar, Paris, France) with a 30-μl loop, using WASP automation (Copan Diagnostics). After inoculation and streaking of the specimens onto BAPs, a single bacitracin disk was dispensed onto each BAP by the WASP instrumentation. All plates were incubated in CO_2_ in the WASPLab system (Copan Diagnostics) for 24 h before images were taken for analysis.

**FIG 1:**
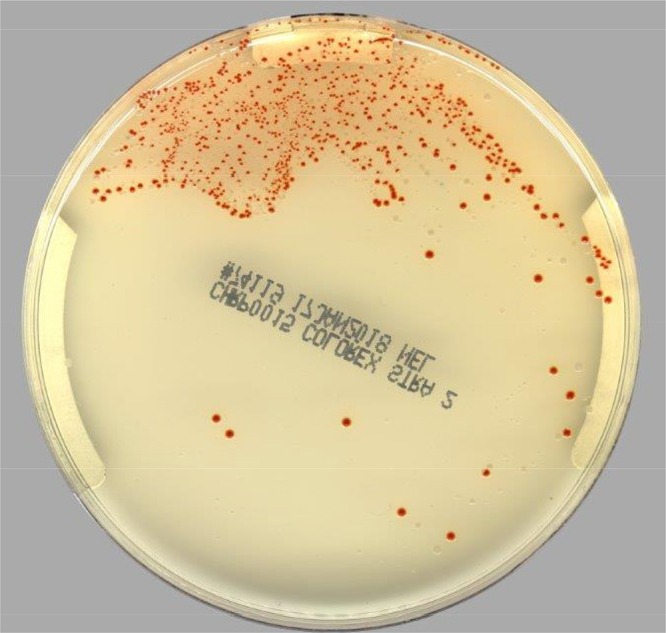
Example of Colorex Strep A agar growing GAS.

After 24 h of incubation, both plates were examined for the presence of potential GAS. Technologists trained to read chromogenic media and throat cultures for the presence of β-hemolytic colonies were responsible for screening both the Colorex Strep A agar plates and the BAPs. The technologists were blinded to the results of the software analysis prior to reviewing the plates. The presence of orange colonies on the Colorex Strep A agar signified the possibility of GAS (Fig. 1). Other *Streptococcus* species would appear as steel blue or colorless colonies, while growth of other bacteria and yeast would be inhibited. The results from the visual examination of the chromogenic agar by the medical technologist were compared with the results from the PhenoMATRIX CDM software.

### Discrepancy analysis.

Any discrepancies between technologist and PhenoMATRIX CDM image reads were adjudicated by a second technologist image read. Any orange colonies observed by manual image reading of the Colorex Strep A agar were identified using a matrix-assisted laser desorption ionization–time of flight mass spectrometry (MALDI-TOF MS) Biotyper (Bruker Daltonic, Billerica, MA). The performance of the Colorex Strep A agar was also compared to that of BAPs by observing the presence of β-hemolysis and any zone of inhibition around a bacitracin disk (Remel, Lenexa, KS).

Specimens that tested negative by Colorex Strep A agar culture but positive by Lyra PCR were tested a second time at Wisconsin Diagnostic Laboratories (Milwaukee, WI), using the same Lyra Direct Strep PCR assay (Quidel). Repeat testing in an alternative laboratory ensured that testing personnel were fully blinded to any of the other results obtained throughout the study.

### Data analysis.

A composite reference standard was used to define a true-positive result. The composite reference standard consisted of all culture-positive (by either PhenoMATRIX CDM or manual reading) isolates confirmed as GAS by MALDI-TOF MS plus any culture-negative specimens that were confirmed to be positive by both initial and repeat PCR assays. Clinical performance characteristics, including sensitivity, specificity, positive predictive value, and negative predictive value, were calculated.

## RESULTS

Of the 480 pharyngeal specimens included in the study, 96 (20%) were considered true-positive specimens; 84 specimens were culture positive by manual and software image reading and were confirmed to be GAS by MALDI-TOF MS, 3 specimens were negative by manual image reading but positive by two PCR assays and typical orange colonies were detected by software image reading, and 9 specimens were culture negative by both manual and software image reading but positive by two PCR assays, resulting in a total of 96 true-positive specimens for the study. Specifically, 99 specimens were PCR positive for GAS during initial standard-of-care testing. Eighty-one of those 99 specimens were culture positive for GAS by manual and software image reading (confirmed by MALDI-TOF MS identification) and were considered true-positive specimens. Of the remaining 18 PCR-positive specimens, 12 were positive by repeat PCR testing and were considered true-positive specimens (3/12 specimens showed orange colonies by software image reading but were culture negative by manual image reading, and 9/12 specimens were culture negative by both manual and software image reading). The last 6 specimens were considered negative, because they were culture negative by both manual and software image reading and negative by repeat PCR testing. Lastly, 3 specimens were PCR negative but culture positive on chromogenic agar by both manual and software image reading, with confirmation as GAS by MALDI-TOF MS. Although these 3 specimens were not retested in a second Lyra PCR assay, according to our definition they were considered true-positive specimens, because they were confirmed positive cultures.

**FIG 2:**
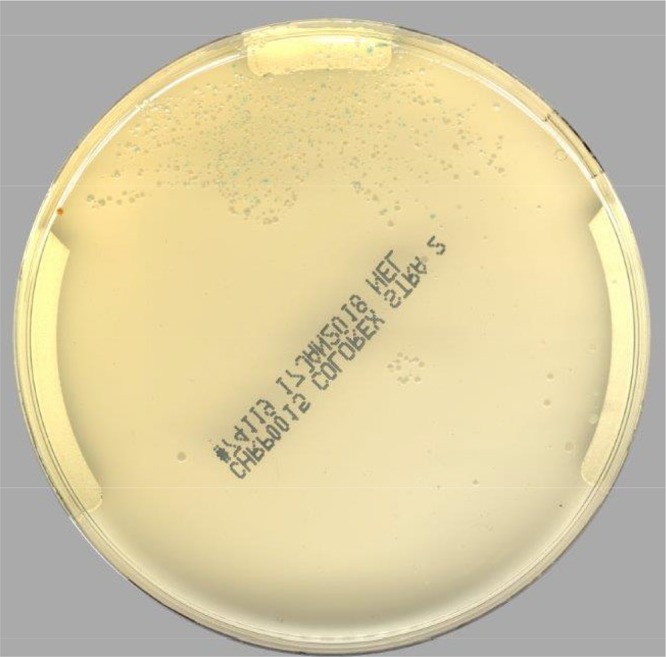
Example of orange colonies growing on Colorex Strep A agar that were not confirmed as GAS.

Table 1 shows the sensitivity and specificity, compared to true-positive specimens, of the five different approaches, namely, Lyra molecular assay, technologist manual reading of the Colorex Strep A agar images, PhenoMATRIX CDM reading of the Colorex Strep A agar images, manual detection of β-hemolytic colonies on BAP images, and manual detection of β-hemolytic colonies on BAP images accompanied by any zone of inhibition around a bacitracin-impregnated disk. To confirm that the orange colonies growing on chromogenic agar were GAS, MALDI-TOF MS was performed for each suspected colony, as identified by both manual and PhenoMATRIX CDM image reading. All 93 cultures that were interpreted as orange colonies were able to be tested by MALDI-TOF MS. Of these, 84 cultures (90.3%) were confirmed as GAS by MALDI-TOF MS. The remaining 9 isolates were identified as Kocuria rhizophila (1 isolate), Staphylococcus aureus (1 isolate), Staphylococcus simulans (1 isolate), Lactobacillus rhamnosus (1 isolate), Rothia mucilaginosa (1 isolate), Streptococcus salivarius (2 isolates), and S. agalactiae (2 isolates). All 9 false-positive cultures were interpreted as orange colonies by both manual and software image reading (an example is shown in Fig. 2). The 1 culture that grew orange colonies identified as S. aureus was also PCR positive; based on these results, it was classified as false positive by culture, since no GAS isolates were identified among the orange colonies, but true positive by PCR. An additional 14 specimens were considered false positive by PhenoMATRIX CDM reading alone; 6 were described as light brown colonies upon manual reading, and the remaining 8 were related to residual matrix/residual sample showing some coloration.

**TABLE 1:** Sensitivity and specificity, compared to true-positive specimens, for the five methods studied

Method	Sensitivity (%) (no. positive/total no.)	Specificity (%) (no. positive/total no.)	Positive predictive value (%)	Negative predictive value (%)
Lyra molecular assay	96.9 (93/96)	100 (384/384)	100	99.2
Manual reading of Colorex Strep A agar images	87.5 (84/96)	97.7 (375/384)	90.3	96.9
PhenoMATRIX reading of Colorex Strep A agar images	90.6 (87/96)	94.0 (361/384)	79.1	97.6
Manual detection of β-hemolytic colonies on BAP images	83.3 (80/96)	69.3 (224/384)	44.7	93.3
Manual detection of β-hemolytic colonies on BAP images with any zone of inhibition with bacitracin disk	39.5 (38/96)	83.1 (319/384)	36.9	84.6

**FIG 3:**
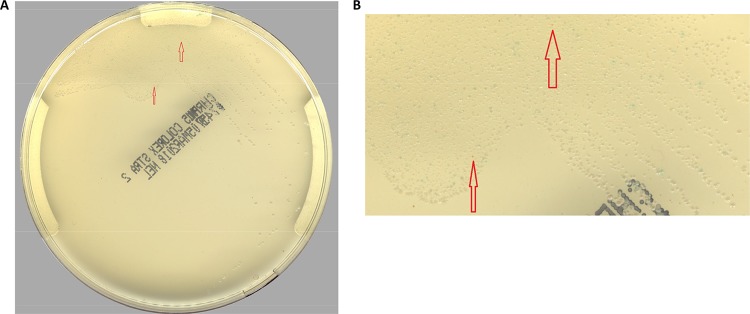
Example of very small, buried colonies in the first quadrant of group A streptococci that were detected only by the software. (a) Whole plate, with arrows pointing to two small orange colonies. (b) Magnified area (×4 magnification), with arrows showing the two orange colonies.

When chromogenic agar plate images were examined manually by a technologist, the sensitivity for detecting orange colonies was 87.5% (84/96 specimens), with a specificity of 97.7% (Table 1), compared to true-positive specimens. When the PhenoMATRIX CDM software was used to screen the chromogenic agar for orange colonies, 3 additional cultures (87/96 specimens) were correctly identified as GAS, resulting in a 3.1% increase in sensitivity (90.6%), compared to manual image reading. The 3 additional specimens that were identified by the software only were confirmed by detection of GAS by PCR twice and thus were considered true-positive specimens. These tiny buried colonies of GAS detected only by the software are illustrated in Fig. 3. Although the small orange colonies were in heavy growth, they were identified by the software as potentially culture positive, based on features such as the correct color and hue. Secondary image review did not categorize the colonies as the wrong color or as a residual matrix or residual specimen. Although these small colonies might not be GAS, it is more likely that they are GAS, especially because the PCR results were positive for GAS. Additional confirmation of the 3 positive specimens by MALDI-TOF MS was not performed, because the software reading of the images was performed after the manual reading had been completed and the agar plates were no longer available.

In a comparison of only the results of the software interpretations versus technologist manual reading of images for the detection of orange colonies, manual image reading detected 93 of the 110 (84.5%) positive specimens detected by the PhenoMATRIX CDM software. In contrast, none of the 361 cultures interpreted as negative by the software was found to be positive by manual image reading. Manual reading from BAPs for β-hemolysis and/or a zone of inhibition around a bacitracin disk were suboptimal, with sensitivities and specificities of 83.3% and 69.3% and of 39.5% and 83.1%, respectively (Table 1).

## DISCUSSION

Two recently published studies demonstrated the ability of the PhenoMATRIX CDM software to sort positive and negative cultures based on pigmentation production on chromogenic media (8, 9). One study was a four-center study that utilized four different chromogenic agars and tested over 57,000 MRSA screening cultures. That study showed 100% sensitivity of the PhenoMATRIX CDM software in comparison with manual reading of culture images, meaning that the software never called a screening culture negative that manual reading called positive. In fact, that study indicated that the software was able to detect an additional 153 true-positive specimens that were missed by manual reading (9). Similarly, a second investigation, which was a three-center study with two chromogenic media for vancomycin-resistant enterococci that reviewed over 100,000 screening cultures, also showed 100% sensitivity of the PhenoMATRIX CDM software in comparison with manual image reading (8). In that study, the software was able to detect nearly 500 additional true-positive cultures that were missed by manual reading.

The current study utilized the PhenoMATRIX CDM software with this novel chromogenic agar for the detection of GAS in throat cultures from pediatric patients who presented to the emergency department of a freestanding pediatric medical center. We showed that the trained software not only was able to detect all GAS-positive cultures identified by manual image reading but also was able to detect 3 additional true-positive cases that were missed by manual image reading. This study also demonstrated that the Colorex Strep A agar, when used with the PhenoMATRIX software, had very good sensitivity and specificity (90.6% and 94.0%, respectively), compared to an FDA-approved PCR assay for GAS (96.9% and 100%, respectively). Preliminary data by Gaskin et al. revealed similar findings (sensitivity of 96.7% and specificity of 100%) when Colorex A Strep A agar was compared to loop-mediated isothermal amplification (LAMP) for the detection of GAS (11). Another study reported a sensitivity of 100% and a specificity of 97.45% when the agar was used in conjunction with the PhenoMATRIX CDM software, compared to manual reading of BAPs with sulfamethoxazole and trimethoprim, for GAS (12).

The Colorex Strep A agar also showed much better overall performance (whether read by a technologist or by the software), compared to routine cultures plated on BAPs, with or without the use of a bacitracin disk, to facilitate screening for GAS. Our findings are consistent with a previous study that reported superior performance of chromogenic agar and PCR, compared to routine culture, for recovering group B *Streptococcus* (13). In addition, our previous study on the Lyra PCR assay detected GAS in an additional 26 specimens that were culture negative (10). It should be noted that the BAPs used for culture of GAS were not stabbed after inoculation, which might have contributed to the slight decrease in sensitivity observed with screening for β-hemolysis on BAPs. Due to the low sensitivity and specificity of screening for β-hemolysis on BAPs to identify potential GAS isolates, laboratories should not rely on the presence of β-hemolysis, with or without a bacitracin disk, for detection of such pathogens from throat specimens.

Discrepancies in the results of culture and the two PCR assays may indicate true differences in clinical sensitivity and specificity between culture and PCR but also could be due to a limitation of our study. Although the first PCR assay was performed on fresh specimens, the repeat PCR assay was based on samples that had been frozen for up to 45 days. The freezing and thawing of the specimens might have had an effect on the repeat PCR assay and even the culture results. A previous study evaluating the ESwab system for MRSA screening with PCR and culture suggested that, although bacteria frozen in the ESwab system retained viability, this viability might decrease after storage and/or a freeze/thaw cycle (A. Giambra and S. Castriciano, presented at the 2007 European Meeting on Molecular Diagnostics, Scheveningen, The Hague, Netherlands). These findings were similar to those of a study that reported that bacterial quantification was not reliable after freeze/thaw cycles (14). The decreased viability after frozen storage may explain the finding of the initial-PCR-positive but culture-negative samples. One freeze/thaw cycle might also explain the subsequent negative PCR result for the initial-PCR-positive samples, but this is unlikely because nucleic acids have been found to be highly stable in ESwab solution at –70°C for at least 60 days (15). Furthermore, we were able to confirm the two GAS-positive samples that were retested after >12 months. In addition, this would not explain the 3 specimens that originally had negative PCR results that in culture grew GAS that was identified by MALDI-TOF MS. Thus, based on these findings, expanding our definition of true-positive specimens to include confirmed culture-positive specimens plus culture-negative specimens that were PCR positive upon repeat testing seemed prudent.

The PhenoMATRIX CDM software can easily be trained to segregate cultures based on whether they are negative or positive for any specific color, using a variety of chromogenic agars. The use of high-quality image capture allows the software to very accurately determine whether an organism with the color sought is present on the medium after incubation. The advantage of digital software reading, compared to manual reading, is the ability of the software to distinguish differences in pixel color that a highly experienced technologist may find difficult. Digital imaging software demonstrated 100% sensitivity, compared to manual reading, when chromogenic agar was used to identify MRSA (9). Thus, negative cultures (cultures not containing the color sought) can be efficiently grouped together, quickly confirmed as negative by batch visual image inspection, and then batch released by the technologist. Similarly, cultures grouped together as positive can be sent for further identification or confirmation according to each individualized laboratory protocol. Thus, combining culture-interpreting software, such as the PhenoMATRIX CDM software, with chromogenic agars allows for quick and accurate segregation of suspected positive cultures from negative cultures. With future FDA clearance, this software will facilitate negative cultures being automatically reported as negative, without the need for technologist intervention. One limitation of screening for positive specimens using the chromogenic agar is that other bacterial causes of pharyngitis, including group C/G streptococci and Arcanobacterium haemolyticum, are not detected. Thus, laboratories that do screen for these additional pathogens must include a BAP to visualize β-hemolysis.

Since culture is still widely used in clinical laboratories for the diagnosis of GAS, the PhenoMATRIX CDM software in combination with the Colorex Strep A agar could dramatically improve the workflow by reducing turnaround times and hands-on times currently necessary for the reporting of throat culture results, allowing the redirection of laboratory personnel to other, more complex tasks. In addition, the improved sensitivity of the CDM software allows the identification of GAS-positive specimens that are missed by manual technologist review, improving the laboratory diagnosis of pharyngitis caused by GAS.
